# Screening for Cushing Syndrome at the Primary Care Level: What Every General Practitioner Must Know

**DOI:** 10.1155/2017/1547358

**Published:** 2017-07-27

**Authors:** Ernest Yorke, Yacoba Atiase, Josephine Akpalu, Osei Sarfo-Kantanka

**Affiliations:** ^1^Endocrine & Diabetes Unit, Department of Medicine and Therapeutics, School of Medicine and Dentistry, College of Health Sciences, University of Ghana, Legon, Accra, Ghana; ^2^Directorate of Medicine, Endocrine and Diabetes Unit, Komfo Anokye Teaching Hospital, Kumasi, Ghana

## Abstract

Cushing's syndrome is a rare entity, and a high index of suspicion is needed for screening in a primary care setting. The clinical awareness of the primary care physician (PCP) to the highly indicative signs and symptoms such as facial plethora, proximal myopathy, reddish purple striae, and easy bruisability should alert him to look for biochemical evidence of Cushing's syndrome through any of the first-line screening tests, namely, 24-hour urinary free cortisol, overnight dexamethasone suppression test, or late-night salivary cortisol. Commonly used random cortisol measurements are unreliable; hence, general practitioners are encouraged to understand the use of these more reliable tests with increased sensitivity and specificity for screening Cushing's syndrome. In this write-up, we set out to increase awareness about the presentation of Cushing's syndrome and current recommended screening methods as well as their strengths and weaknesses. We relied mainly on the recommendations by the Endocrine Society Guidelines.

## 1. Introduction

Cushing's syndrome (CS) describes the sign and symptom complex due as a result of prolonged supraphysiological levels of circulating glucocorticoid of any type [1]. CS can be exogenous (iatrogenic) or endogenous [1]. Worldwide, exogenous CS represents the bulk of CS which are due as a result of medically prescribed glucocorticoids as well as steroid-containing skin bleaching creams especially in Africa [2–4]. Endogenous CS is diagnosed when there is increased adrenocorticotropic hormone (ACTH) from a pituitary adenoma (Cushing's disease) or an ectopic source as well as an independent adrenal source of cortisol over production [1].

The discussions from here will focus mainly on endogenous CS.

## 2. Epidemiology

In its classic form, CS remains a rare condition associated with diagnostic and therapeutic challenges as well as excess morbidity and mortality [2]. Globally, it has an incidence of 0.2–5 per million/year and a prevalence of 39–79 per million in various populations [5]. The average age-of-onset is 41.4 years with a female to male ratio of 3 : 1. Increased prevalence is seen in those with uncontrolled T2DM, hypertension, or early-onset osteoporosis [5]. Extrapolated estimates suggest an incidence of about 20 per million population in Africa [6].

## 3. Clinical Presentation

Clinical features of CS are multisystemic [7]. The signs and symptoms of CS such as obesity, hypertension, skin changes (plethora, striae, and hirsutism), osteopenia, weakness, psychological problems including depression, menstrual abnormalities, glucose intolerance, impotence, dyslipidaemia, and retarded growth in children which are recognized in over half of the cases of CS are also quite commonly seen in general population, yet CS as an entity is relatively rare [2–8]. Moreover, signs and symptoms of CS vary from patient to patient and the presentation can be cyclic [2, 8].

In pseudo Cushing's syndrome (pseudo-CS), there is physiologic hypercortisolism in the absence of true CS [1, 2]. Such situations may be found in pregnancy, depression, alcohol dependence, obesity, poorly controlled diabetes, physical stress, malnutrition, anorexia nervosa, strenuous exercise, and hypothalamic amenorrhoea; a broader list is presented in Table 1 [1, 2]. The clinical features of pseudo-CS may overlap with CS and also give false positive test results, and so clinicians must be aware to this fact during screening [1, 2, 9]. The rising worldwide prevalence of obesity and clinical features of obesity-related metabolic syndrome typify such overlaps in clinical features as well as difficulties with biochemical diagnosis [10, 11].

Though diagnosing CS is indeed challenging, especially in mild cases, it is extremely crucial because undiagnosed CS increases morbidity (cardiovascular diseases, infection, poor wound healing, osteoporotic fractures, depression, and growth retardation) and mortality [8]. CS remains undiagnosed in significant number of patients with diabetes (2-3%), hypertension (0.5–1%), adrenal incidentalomas (6–9%), and osteoporotic fractures (11%) [2]. The mortality and quality of life associated with CS decrease but not to the population risk after remission of hypercortisolism [7]. The clinical features of CS also regress but may not be complete after successful treatment [12, 13].

Again, the existence of comorbid psychological and psychiatric disorders such as mood disorders and major depression with hypercortisolism states may be as high as 60% in some cases [2, 14]. Other conditions include mania, cognitive impairment, and reduced quality of life [2, 14]. Whilst a bidirectional causal relationship has been suggested in some cases, adequate treatment of the underlying cause of the hypercortisolism may not lead to the complete resolution of these comorbid psychological/psychiatric conditions [14]. It is imperative therefore that these aforementioned associated conditions be sought for and managed appropriately using a multidisciplinary team approach [14].

## 4. Indications for Screening

The Endocrine Society Guidelines [2] recommend screening for CS in patients with the following features:

1. Weight gain and central redistribution of fat
2. Multiple progressive features of CS
3. Unusual features for age (osteoporosis and hypertension in young)
4. Children with retarded growth (decreasing height percentile and increasing weight)
5. Adrenal incidentaloma compatible with adenoma.

## 5. What Are the Recommended Screening Tests?

### 5.1. Ideal Screening Test

An ideal screening test for CS should have high sensitivity without missing mild cases [15]. The test should be simple, affordable, and socially convenient, preferably done without hospitalization, and should be able to differentiate pseudo-CS from true CS with the least number of false positives and negatives [15]. No single test satisfies all these criteria [2].

### 5.2. Screening

Firstly, rule out iatrogenic CS when there is long-term steroid therapy and assess pretest probability of CS based on relatively specific signs [2, 9]. These include proximal myopathy, easy bruisability, purple striae (>1 cm), facial plethora, and, in children, weight gain with decreasing growth velocity [2].

**Figure 1:**
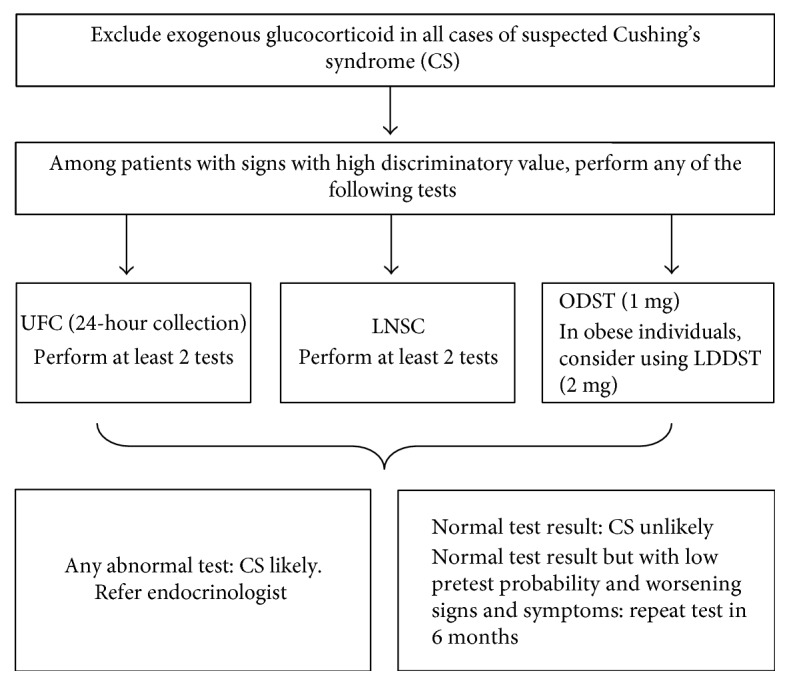
Screening for Cushing's syndrome at the primary care level. CS: Cushing's syndrome; UFC: urinary free cortisol; LNSC: late-night salivary cortisol; DST: overnight dexamethasone suppression test; LDST: low-dose dexamethasone suppression test.

Recommended screening tests and algorithm (Figure 1()) for diagnosis of CS and the principles on which they are based are as follows [2, 16]:

1. 24-hour urinary free cortisol (UFC): there is increased cortisol production with urinary excretion.
2. Late-night salivary cortisol (LNSC): there is loss of diurnal rhythm (not achieving late-night nadir).
3. Dexamethasone suppression tests (DST): there is loss of feedback inhibition on hypothalamic-pituitary-adrenal (HPA) axis.

Dexamethasone-suppressed corticotrophin releasing hormone (CRH) stimulation (Dex-CRH) test may rarely be performed to differentiate pseudo-CS (as in alcoholism, obesity, depression, pregnancy, anorexia nervosa, and uncontrolled diabetes) from true CS [16]. It is described in more detail elsewhere.

Majority of serum cortisol is bound to corticosteroid-binding globulin (CBG) and albumin, and only 4% is free [2]. CBG levels increase in pregnancy and during estrogen therapy, and serum cortisol measurements are unreliable until estrogens are stopped for more than 6 weeks [16]. Salivary and urinary cortisol provides a direct assessment of serum free cortisol as only free cortisol is filtered into saliva and urine [17]. These tests are not affected by conditions that affect CBG levels. Liquid chromatography with tandem mass spectrometry (LC-MS/MS) is the most validated method to measure free cortisol in both saliva and urine as immunoassays such as radioimmunoassay (RIA) and enzyme-linked immunosorbent assay (ELISA) tend to give false-positive results [9, 18].

## 6. Measurements

### 6.1. 24-Hour Urinary Free Cortisol (UFC)

UFC was earlier considered as the gold standard [16]. UFC has the advantage of detecting hypercortisolism in situations with altered CBG levels. However, its sensitivity and specificity are lower than other tests and hence mild CS can be missed [16]. Moreover, it gives false positive and negative results in many conditions and with many drugs [17]. High fluid intake, incomplete collection, contamination, and decreasing GFR (<60 ml/min) can make UFC unreliable [17]. Interestingly, in a study by Ceccato et al. published in 2015 [19], they found out that UFC alone performed as well as using a paired combination of either UFC, DST, and LNSC or all three tests combined. They concluded that among patients with suspected hypercortisolism, UFC measured by LC-MS/MS achieves the best diagnostic accuracy [19]. UFC is unacceptable and inconvenient for many patients, and it cannot assess loss of circadian rhythm, which occurs early in CS [17]. UFC should ideally be repeated because of variability in cortisol excretion. Diagnostic criteria for Cushing's syndrome using UFC are suggested by a value greater than the normal range for the assay [2]. The diagnostic accuracy can be improved by measuring urinary creatinine at the same time. Incomplete collection of urine is deemed to have occurred, and urine collection must be repeated when creatinine levels are <1.5 g per day for men and <1 g per day for women [10].

### 6.2. Late-Night Salivary Cortisol (LNSC)

Cortisol secretion is pulsatile, with maximum secretion in early morning and minimum at midnight (11 PM–2 AM) [20]. Any change in serum cortisol levels is immediately reflected in saliva [7]. Late-night serum and salivary cortisol rely on the fact that patients with CS have loss of circadian rhythm with lack of late-night cortisol nadir [20]. Obtaining saliva sample during bedtime is easy, noninvasive, and stress free and can be done at home. Collected saliva can be stored in a refrigerator for 7 days and sent by mail to the laboratory at room temperature [20]. LNSC is not reliable in patients with disturbed sleep, shift work, smoking, chewing tobacco, brisk brushing of teeth, depression, and critical illness [20]. Late-night salivary cortisol greater than 145 ng/dl (4 nmol/liter) suggests CS [2]. Because of variability in cortisol secretion, LNSC should be repeated [2].

### 6.3. Overnight and Low-Dose Dexamethasone Suppression Tests (ODST and LDDST)

Dexamethasone suppression test (DST) assesses loss of feedback inhibition of CRH and ACTH secretion [16]. In ODST, which is a screening test for hypercortisolism, 1 mg dexamethasone is taken orally at 11 PM. Serum cortisol levels are checked between 8-9 AM next morning [21]. In children, 0.3 mg/m^2^ surface area of dexamethasone is used [22]. In LDDST, which is a confirmatory test for hypercortisolism, 0.5 mg dexamethasone is taken every 6 hours for 2 days (starting from 9 PM). Serum cortisol is checked at the beginning and at the end. With ODST and LDDST, serum cortisol greater than 1.8 *μ*g/dl (50 nmol/liter) after dexamethasone suppression suggests the diagnosis of CS [2].

Increase in CBG will lead to false positive whereas a decrease in CBG will give false-negative tests results [16]. Exercise and poor sleep after dexamethasone will lead to false positivity [16]. Enzyme inducers, gastrointestinal malabsorption, and rapid transit time decrease the available drug for suppression and may lead to false positive results [16]. Enzyme inhibitors and mild hypercortisolism cause false-negative response [15]. Dexamethasone dose may need modification in obese individuals, and it has been suggested that the false-positivity rate decreases from 8% to 2% with 2 mg dexamethasone rather than 1 mg [23]. In a recent review by Loriaux in 2017 [10], he suggests that in populations with a high prevalence of obesity such as the United States of America, the positive predictive value of the ODST is only 0.4% and therefore discourages its use in diagnosing CS.

## 7. Further Comments

Three tests, which can be performed easily in primary care, are UFC, LNSC, and ODST. Whilst the Endocrine Society Guidelines contends that there is no single best test, LNSC is the current screening test of choice for majority of cases [8]. At least two first-line tests should be abnormal to diagnose CS [2].

LNSC has higher sensitivity and specificity when compared to UFC, but the prerequisite is that patient should maintain regular diurnal lifestyle and avoid tobacco chewing, smoking, vigorous brushing, and topical oral steroid preparation [2, 8]. Again, several meta-analyses have shown that LNSC has comparable efficiency to UFC and LDDST and they perform well in both outpatient and inpatient settings [24–26]. Moreover, LNSC can diagnose mild CS in 17.3% of patients with normal/near-normal UFC [27]. As pseudo-CS patients maintain intact circadian rhythm, LNSC remains true negative in them whereas UFC shows false positivity [20]. Also, after pituitary surgery for Cushing's disease, disease recurrence is picked up early by LNSC than by UFC [17]. Due to these favorable characteristics, LNSC is currently preferred over UFC. It must be stated however that LNSC tests are not routinely available at the primary care level.

In practice, tests used in diagnosing CS vary across different countries and geographical areas of the world and they partly differ from the currently available guidelines [28]. Valassi et al. analyzed the data on the diagnostic tests performed in 1341 patients with Cushing's syndrome (CS) who have been entered into the European Registry on Cushing's syndrome (ERCUSYN) database between January 1, 2000 and January 31, 2016 from 57 centers in 26 European countries [28]. They found out that of the first-line tests, UFC test was performed in 78% of patients, DST in 60%, and LNSC in 25%. This may be partly due to the differences in the availability of the different tests in different countries, and therefore there may be the need for harmonization of guidelines [28].

It is recommended that when there is high pretest probability, patients with a normal test result should be referred to the endocrinologist for further assessment [2]. However, when the results are normal but the pretest probability is low, the tests should be repeated in 6 months if symptoms and signs progress [2, 15].

Whilst the use of random serum cortisol is high among many general practitioners, using random cortisol or plasma ACTH to screen for CS is unreliable and therefore not recommended [2, 15].

## 8. Further Tests

It is recommended that an endocrinologist should choose any of these second-line tests. Any positive test should be confirmed with any of the recommended screening tests above (i.e., UFC, LNSC, and ODST) or with dexamethasone-suppressed corticotrophin releasing hormone (CRH) stimulation test [2]. Midnight serum cortisol can also be done [2]. The latter two tests are described briefly below.

### 8.1. Dexamethasone-CRH (Dex-CRH) Test

This test combines a LDDST and CRH stimulation test to differentiate pseudo-CS from true CS. True CS patients respond to CRH injection with increases in cortisol level even when pretreated with dexamethasone. 0.5 mg of dexamethasone is given every 6 hours starting from about 12 noon with the administration of CRH 2 hours after the last dose of dexamethasone. Plasma cortisol and ACTH are measured every 15 minutes for one hour [29]. A serum cortisol value >38 nmol/l or 1.4 *μ*g/dl fifteen minutes after CRH administration suggests CS. A pseudo-CS patient does not respond to CRH injection which is thought to be due to chronic CRH stimulation hence the blunted response [16, 29]. The sensitivity and specificity of this test is 90–100% and 67–100%, respectively [29].

### 8.2. Midnight Serum Cortisol

Collecting serum sample for late-night serum cortisol requires admission for 48 hours. After 48 hours or more of inpatient admission, “sleep” blood samples must be taken within 5–10 minutes after waking and for “awake” samples, through an indwelling line to avoid false positive results [30]. “Sleeping” or “awake” midnight serum cortisol of less than 207 nmol/l (7.5 *μ*g/dl) is a reasonable cut-off to exclude the diagnosis of CS [31].

## 9. Screening Tests in Special Circumstances

Patients with adrenal incidentalomas do not have consistently high urinary and salivary cortisol and are therefore better screened by DST [2, 27]. When CS is suspected clinically but laboratory results are normal, cyclic-CS should be suspected and confirmed by doing UFC or LNSC during symptomatic phase [26] (Table 2). In normal pregnancy, UFC excretion is normal in the first trimester but increases up to 3-fold by term to values seen in women with Cushing's syndrome [32]. Therefore, during the second or third trimester, only UFC values greater than 3 times the upper limit of normal should indicate Cushing's syndrome [2, 32]. Patients with chronic kidney disease should be screened for CS using DST or LNSC as UFC measurement are unreliable [2].

## 10. Further Steps and Localization

The primary care doctor should promptly refer all positive cases after initial screening tests.

Once diagnosis of CS is made, the next step is to determine the cause. The localization of the source of hypercortisolism should ideally be done by an endocrinologist or a specialist physician with interest in endocrine disorders [2].

Serum ACTH level will be measured to see if the disease is ACTH-independent or ACTH-dependent [5]. If ACTH level is suppressed, then an adrenal cause is sought by MRI or CT scan of the abdomen. If ACTH level is high, the patient will have additional tests to determine if there is a pituitary adenoma or ectopic tumor [2, 5].

## 11. Limitations

It must be recognized that in practice, there is no universally agreed approach in screening for CS. As stated earlier, analysis of confirmed cases of CS captured in a European registry revealed varied use of screening tests across the 26 countries involved [28]. On a worldwide level, access to these variously suggested screening tests and payment mechanisms available vary tremendously.

## 12. Conclusion

Cushing's syndrome is a rare entity, and in a primary care setting, a high index of suspicion is needed for screening. The clinical awareness of the primary care physician to the highly indicative signs and symptoms such as facial plethora, proximal myopathy, reddish purple striae, and easy bruisability should alert him to look for biochemical evidence of CS through any of the first-line screening tests, that is, 24-hour UFC, ODST, or late-night salivary cortisol.

Most often in clinical practice, LNSC is preferred because of its higher sensitivity and specificity compared to the others though strict precautions should be followed prior to sampling such as avoiding smoking, tobacco, brisk brushing of teeth, and irregular sleep patterns. It is recommended to combine more than one investigation based on the patient's presentation and medical history. Any abnormal finding can then be combined with a low-dose DST, and if that also turns out positive, further screening and referral to an endocrinologist should be done to localize the source.

As each investigation has its strengths and weaknesses in different circumstances and presentations, the clinical judgment of the clinician is of extreme importance in screening for CS underlining the need for proper awareness and sensitization of the PCPs to the existence of this rare though potentially treatable disorder. Not withstanding these recommendations, due to differences in local guidelines and availability of these tests across geographical regions of the world, the PCP must tailor his/her request to reflect local reality.

## Figures and Tables

**Table 1:** Conditions suggested by the Endocrine Society that may present as pseudo Cushing's syndrome [2].

Physiologic hypercortisolism in the absence of Cushing's syndrome
May have Cushing's syndrome features
Pregnancy
Depression
Other psychiatric diseases
Alcohol dependence
Glucocorticoid resistance
Morbid obesity
Uncontrolled diabetes
May not have Cushing's syndrome features
Physical stress such as hospitalization, surgery, and pain
Malnutrition
Anorexia nervosa
Vigorous chronic exercise
Hypothalamic amenorrhea
CBG excess (serum cortisol increase; not UFC)

CBG: cortisol-binding globulin; UFC: urinary free cortisol.

**Table 2:** Preferred screening tests in special circumstances [2, 9, 15].

Situations	Test preferred	Test not preferred
Cyclic Cushing's syndrome [21]	UFC or LNSC	DST
Mild Cushing's syndrome [21]	LNSC or DST	UFC
Pseudo Cushing's syndrome [21]	LNSC or DST	UFC
CS patients on antiepileptics [21]	UFC or LNSC	DST
Adrenal incidentaloma [21]	DST	UFC or LNSC
Pregnancy [21]	UFC	DST, LNSC
Severe chronic kidney disease [21]	DST or LNSC	UFC
Low pretest probability [15]	UFC	
High pretest probability [15]	LNSC	

UFC: urinary free cortisol; LNSC: late-night salivary cortisol; DST: dexamethasone suppression test.

## Abbreviations

ACTH: Adrenocorticotropic hormone
CBG: Corticosteroid-binding globulin
CRH: Corticotropin-releasing hormone
CS: Cushing's syndrome
CVD: Cardiovascular disease
Dex-CRH test: Dexamethasone-suppressed CRH stimulation test
DST: Dexamethasone suppression test
ELISA: Enzyme-linked immunosorbent assay
HPA: Hypothalamic-pituitary-adrenal axis
GFR: Glomerular filtration rate
LC-MS/MS: Liquid chromatography with tandem mass spectrometry
LNSC: Late-night salivary cortisol
RIA: Radioimmunoassays
T2DM: Type 2 diabetes mellitus
ULN: Upper limit of normal
UFC: 24-hour urinary free cortisol.
